# Pharmacomicrobiomics in precision cancer therapy: bench to bedside

**DOI:** 10.3389/fimmu.2024.1428420

**Published:** 2024-09-09

**Authors:** Khanh Le Ngoc, Tran Thu Ha Pham, Tiep Khac Nguyen, Phung Thanh Huong

**Affiliations:** Faculty of Biotechnology, Hanoi University of Pharmacy, Hanoi, Vietnam

**Keywords:** cancer therapy, microbiota, microbiome intervention, pharmacomicrobiome, precision medicine

## Abstract

The burgeoning field of pharmacomicrobiomics offers promising insights into the intricate interplay between the microbiome and cancer, shaping responses to diverse treatment modalities. This review aims to analyze the molecular mechanisms underlying interactions between distinct microbiota types and cancer, as well as their influence on treatment outcomes. We explore how the microbiome impacts antitumor immunity, and response to chemotherapy, immunotherapy, and radiation therapy, unveiling its multifaceted roles in cancer progression and therapy resistance. Moreover, we discuss the challenges hindering the development of microbiome-based interventions in cancer therapy, including standardization, validation, and clinical translation. By synthesizing clinical evidence, we underscore the transformative potential of harnessing pharmacomicrobiomics in guiding cancer treatment decisions, paving the way for improved patient outcomes in clinical practice.

## 1 Introduction

Precision medicine, or personalized therapy, is a rapidly evolving trend in modern healthcare, tailoring treatment to individual patients by selecting the right drug, at the right dose and at the right time based on their specific cellular, molecular, and genetic characteristics. This approach optimizes treatment effectiveness while minimizing the risk of adverse drug reactions (ADRs). Among therapeutic fields, cancer treatment has seen particularly swift advancements in precision medicine due to its typical characteristics such as the highly heterogeneous nature of tumors, substantial variability in drug response among individuals, and the significant side effects associated with cancer therapies (1). Additionally, the high costs of treatment and the invaluable cost of life opportunities necessitate precise treatment protocols without room for trial and error. Biomarkers used to predict treatment response and disease prognosis not only aid in selecting appropriate drugs for patients but also drive innovation in drug development, reducing time and costs for clinical trials and substantially increasing success rates (2).

To date, the most extensively utilized biomarker in personalized cancer therapy is the somatic mutations found in tumors. These mutations assist in identifying patients who are likely to respond favorably to targeted therapy and immunotherapy. Furthermore, the distinctive germline mutations present in individual patients significantly influence the pharmacokinetics and pharmacodynamics of chemotherapy, enabling the prediction of those at risk of experiencing severe ADRs (3, 4). As a result, pharmacogenomics has emerged as a rapidly advancing field, essential for integrating personalized cancer therapy into clinical practice. However, clinical studies and trials have indicated that pharmacogenomic biomarkers only partially account for the inter-individual variation in drug response (3, 5). Therefore, to fully leverage the benefits of personalized therapy for patients, additional research is necessary to investigate other types of biomarkers that can help elucidate the differences among individual patients with impacts on treatment response.

Although the role of human microbiome in health and various diseases, including cancer, has been known for a long time, the concept of Pharmacomicrobiomics only emerged around 2010 as an extension of Pharmacogenomics (6). The human microbiome refers to the community of microorganisms residing in specific body environments, with the gut microbiome being the first and most extensively studied (7). However, nowadays, increasing attention is being paid to the important role of microbiota in other body environments such as the skin microbiome, vaginal microbiome … and even more recently, microbiota discovered within the microenvironments of tumors (6). Pharmacomicrobiomics is a field that investigates the interaction between individual’s microbiomes and drug response to understand how the composition and activity of these microorganisms influence the pharmacokinetics and pharmacodynamics of various medications (8). Pharmacomicrobiomics explores how differences in the microbiome among individuals can affect drug metabolism, efficacy, and toxicity, ultimately influencing an individual’s response to treatment. This field has implications for personalized medicine, as it may help optimize drug therapy by considering an individual’s microbiome profile.

There have been numerous studies on the role of various human microbiomes in tumorigenesis and progression across different cancer types (9–14). However, only in recent years have scientists begun to explore the influence of the microbiome on cancer treatment response (15). With the increasing demand for the rapid adoption of personalized medicine in cancer therapy to prolong survival and improve the quality of life of patients, pharmacomicrobiomics may contribute vital biomarkers to enhance the translation of precision medicine into clinical practice. This review analyzes the molecular mechanisms of interaction between different microbiome types and tumors, as well as their response to various cancer treatment modalities. It also analyzes the challenges to consider in developing this application direction, proposes potential solutions to benefit patients, and ultimately provides clinical evidence for the advancement of pharmacomicrobiomics in practice.

## 2 The role of microbiome in cancer

### 2.1 The microbiota dysbiosis in cancer development and progression

There is compelling evidence indicating a correlation between various types of human microbiomes and different types of cancer (16). Among these, the gut microbiome has been the first and most extensively studied. It is well-documented that the gut microbiome influences systemic metabolic balance and immune function, thus playing a significant role in the tumorigenesis and progression of various cancer types, from gastrointestinal cancers to breast or lung cancer (**Table 1**). Additionally, changes in the bacterial ecosystem on the skin, which is the body’s largest organ, also have implications for breast health and the risk of developing breast cancer (10). Reduced alpha diversity within the oral microbiome could potentially correlate with an elevated likelihood of developing lung cancer, offering a potential indicator for predicting lung cancer risk (11). In particular, recent discoveries regarding the existence of intratumoral microbiota also underscore their significant influence on tumor development, invasion, and metastasis (12, 56).

**Table 1:** Impacts of microbiome reprogramming on cancer hallmarks.

Cancer type	Subjects	Change in Microbiome Composition and Function	Cancer Hallmarks affected	Ref
CRC	Mouse model and CRC patients.	- *Peptostreptococcus anaerobius* is significantly enriched in patients with CRC. - IDA treatment or implantation of *P. anaerobius* promotes CRC progression in both xenograft model and ApcMin/+ mice.	Metabolic reprogramming: Colorectal carcinogenesis is promoted by trans-3- indoleacrylic acid (IDA), a tryptophan metabolite originating from *Peptostreptococcus anaerobius*.	(13)
CRC	CRC patients and healthy controls.	EO-CRC showed a tendency to be linked with an abundance of *Flavonifractor plauti.*	Metabolic reprogramming: The multiomics signatures of EO-CRC indicated an inclination towards increased *Flavonifractor plauti* levels and heightened metabolism of tryptophan, bile acid, and choline.	(17)
CRC	CRC patients and healthy controls.	In the CRC group, the microbiome exhibited a notable enrichment of strains from *Bifidobacterium*, Bacteroides, and Megasphaera, while the healthy control group showed higher abundance of *Collinsella, Faecalibacterium*, and *Agathobacter* strains.	Genome instability: The KRAS mutant type demonstrated a close association with *Faecalibacterium, Roseburia, Megamonas, Lachnoclostridium*, and *Harryflintia*. KRAS mutations displayed a negative correlation with the presence of *Bifidobacterium* and a positive correlation with *Faecalibacterium*.	(18)
CRC	CRC and colorectal adenoma patients.	Eight gut microbiome-associated serum metabolites (GMSM panel) were significantly changed in both CRC and adenoma.	Metabolic reprogramming: The gut microbiome alterations observed in individuals with CRC are linked to changes in the serum metabolome.	(14)
CRC	Human cell and mouse model.	The capability of pks+ *E. coli* to create colibactin (DNA adducts) in mammalian cells and mice provides further evidence supporting colibactin's role in cancer development or progression.	Genome instability:DNA double-strand breaks (DSBs) by producing colibactin, a small-molecule genotoxin that causes alkylation of DNA through an unusual electrophilic cyclopropane mechanism.	(19)
CRC	Colorectal neoplasia patients and individuals undergoing outpatient colonoscopy.	*Enterotoxigenic Bacteroides fragilis* (ETBF) produces the bft toxin, which was identified in a majority of surgically resected tumors, with consistent presence in late-stage CRCs (CRCs).	Inflammation and Genome instability: Exposure to bft in the human colon might induce chronic, potentially focal, mucosal inflammation, creating sites susceptible to DNA mutagenesis and the development of cancer.	(20)
CRC	CRC patients and healthy subjects. Mouse model.	- Fusobacterium spp. show increased presence in human colonic adenomas relative to surrounding tissues and in stool samples from colorectal adenoma and carcinoma patients compared to healthy subjects. - In the Apc(Min/+) mouse model that develops intestinal tumors, Fusobacterium nucleatum enhances the number of tumors and specifically attracts myeloid cells infiltrating the tumors, potentially accelerating tumor advancement.	Inflammation: *Fusobacteria* contribute to the development of a proinflammatory microenvironment by attracting immune cells that support the progression of colorectal neoplasia.	(21)
BC	Mouse model Cell lines. BC patients and healthy women.	- Cadaverine treatment of Balb/c female mice (500 nmol/ kg p.o. q.d.) grafted with 4T1 breast cancer cells ameliorated the disease. - In breast cancer cell lines, cadaverine within its physiological serum range (100–800 nM) reversed endothelial-to-mesenchymal transition, inhibited cell migration and invasion. - The abundance of *Escherichia coli* CadA and also * Escherichia coli, Enterobacter cloacae* and *Hafnia alvei* LdcC DNA slightly decreased in BC patients. The decline in CadA and LdcC abundance was notably more significant in clinical stage 0 patients as compared to the pool of all patients. The fecal samples from stage 1 patients revealed substantially lower * Escherichia coli* LdcC protein levels in comparison to those observed in healthy women.	Metabolic reprogramming: Women with early-stage BC, versus control women, had reduced abundance of the CadA and LdcC genes in fecal DNA, both responsible for bacterial cadaverine production, a metabolite of the microbiome which reduces BC aggressiveness through trace amino acid receptors.	(22)
BC	Premenopausal BC patients and premenopausal healthy controls.	Between postmenopausal patients and postmenopausal controls, 45 species exhibited significant differences. In postmenopausal patients, 38 species, including *Escherichia coli, Klebsiella sp_1_1_55*, *Prevotella amnii, Enterococcus gallinarum, Actinomyces sp*. *HPA0247, Shewanella putrefaciens*, and *Erwinia amylovora*, were enriched, while 7 species, such as *Eubacterium eligens* and *Lactobacillus vaginalis*, were less abundant.	Immunity: *Acinetobacter radioresistens* and *Enterococcus gallinarum* showed weak positive associations with high-sensitivity C-reactive protein expression, while *Actinomyces sp. HPA0247* was weakly negatively correlated with CD3+CD8+ T cell numbers. Metabolic reprogramming:* Shewanella putrefaciens* and *Erwinia amylovora* displayed weak positive associations with estradiol levels.	(23)
BC	Postmenopausal BC patients and age-matched controls with normal mammograms.	BC cases had significant oestrogen-independent associations with the IgA-positive and IgA-negative gut microbiota.	Metabolic reprogramming: Gut microbiota may impact BC risk through modifications metabolism, oestrogen recycling, and immune pressure.	(24)
BC	Cell lines, mouse model and BC patients and healthy subjects.	- In experiments with mice and in laboratory studies, lithocholic acid (LCA), a secondary bile acid, decreased cancer cell proliferation and VEGF production, as well as reduced the aggressiveness and metastatic potential of primary tumors. - BC patients reduced abundance of baiH in *Clostridium sordelli, Pseudomonas putida*, and *Staphylococcus aureus*. In early-stage BC patients, a more significant decline in the abundance of baiH in *Bacteroides thetaiotaomicron* and *Pseudomonas putida* was observed..	Metabolic reprogramming: Comparing all patients to healthy controls, BC patients had lower levels of baiH in *Clostridium sordelli, Staphylococcus haemolyticus, Escherichia coli*, and * Pseudomonas putida*, aligning with decreased Lithocholic acid (LCA), a bacterial metabolite. In the early stages of BC, there was a notable decrease in LCA biosynthesis and levels, suggesting a potential role for this pathway in human disease through the activation of the TGR5 receptor.	(25)
LC	LC patients and healthy volunteers.	The abundance of 232 operational taxonomic units (OTUs) showed significant differences between the Healthy control and Lung cancer groups.	Metabolic reprogramming: Through a combined analysis, associations were observed between lung cancer (LC)-associated microbes and metabolites. Notably, *Erysipelotrichaceae_UCG_003*, * Clostridium*, and *Synergistes* were correlated with glycerophospholipids.	(26)
LC	Early-stage LC patients and healthy individuals.	Species more abundant in the cancer group were primarily from the *Bacteroides* and *Proteobacteria* phyla. Conversely, species exhibiting a significant decrease in the cancer group were mainly from the *Firmicutes* and *Actinobacteria* phyla.	Metabolic reprogramming: The cancer group exhibited enrichment in pathways associated with cellular antigens, steroid biosynthesis, ubiquitin system, transcription-related proteins, bile secretion, and fatty acid elongation in mitochondria. On the other hand, pathways related to bacterial motility proteins, bacterial chemotaxis, flavone and flavonol biosynthesis, apoptosis, and G protein-coupled receptors showed a decrease in the cancer group.	(27)
LC	LC patients and healthy controls.	Healthy controls exhibited a higher abundance of the bacterial phylum *Actinobacteria* and the genus *Bifidobacterium*, whereas patients with LC demonstrated elevated levels of *Enterococcus*. A notable decline in the normal function of the gut microbiome was observed in LC patients.	Metabolic reprogramming: There was a significant decline in the functional abundance spectrum, including 24 gut microbiota metabolic pathways in LC patients. This decline included a reduction of more than 80% in the expression of functional proteins involved in chromatin structure and dynamics, as well as RNA processing and modification. Conversely, there was an increase of over 10% in protein expression levels related to extracellular structures in the metabolic functions associated with LC patients.	(28)
LC	LC patients and healthy controls.	The LC group had elevated levels of *Bacteroides, Veillonella*, and *Fusobacterium*, but lower levels of *Escherichia-Shigella, Kluyvera, Fecalibacterium, Enterobacter*, and *Dialiste*r compared to the healthy control group.	Inflamation: *Escherichia-Shigella* and *Enterobacter* showed positive correlations with serum NLR levels.Dialister displayed negative correlations with serum levels of NLR and PLR. Additionally, correlations were identified between *Dialister* and serum levels of IL-12 and sCTLA-4.	(29)
CC	Cervical cancer patients and healthy controls.	In patients with cervical cancer (CC), there was a notably higher proportion of the *Proteobacteria* phylum. Seven genera exhibited significant differences in relative abundance between CC and controls, including *Escherichia-Shigella*, *Roseburia, Pseudomonas, Lachnoclostridium, Lachnospiraceae_UCG-004, Dorea*, and *Succinivibrio.*	Inflamation: Bacterial microbiome-induced tumorigenesis is believed to be associated with an inflammatory response mediated by MAMP and their activation of PRRs. This activation induces the transcription of antibacterial proteins through an intracellular signaling cascade in the host epithelial cell. Additionally, pro-inflammatory cytokines such as IL-17, TNF-α, and IFN-γ were upregulated. Metabolic reprogramming: The gut microbiota play a role in modulating the enterohepatic circulation of estrogens, which circulate to exert effects on target organs like the breast and uterine cervix.	(30)
PC	Patients with benign prostatic conditions or intermediate or high-risk clinically localized prostate cancer.	In prostate cancer cases, a higher relative abundance of *Bacteroides massiliensis* was observed compared to controls. *Faecalibacterium prausnitzii* and *Eubacterium rectalie* had a higher relative abundance among controls.	Metabolic reprogramming: *Faecalibacterium prausnitzii* plays a crucial role in the metabolism of acetic acid, which can subsequently be converted into butyric acid. Butyric acid, the most abundant short-chain fatty acid (SCFA) in the colon, is recognized for its anti-tumor activities, primarily characterized by inducing apoptosis and diminishing proliferation. Additionally, a deficiency of *F. prausnitzii* has been observed in patients with Crohn's disease.	(31)
LiC	NAFLD patients and healthy subjects.	In the gut microbiota of healthy subjects, five genera, including *Alistipes* and *Prevotella*, were significantly more abundant compared to Non-alcoholic fatty liver disease (NAFLD) patients. Conversely, NAFLD patients showed increased levels of *Escherichia, Anaerobacter, Lactobacillus*, and *Streptococcus* in their gut microbiota compared to healthy subjects.	Inflamation: The dysbiosis of the gut microbiota, along with gut microbiota-mediated inflammation of the intestinal mucosa, was evident in NAFLD patients. This inflammatory response was characterized by decreased numbers of CD4+ and CD8+ T lymphocytes and increased levels of TNF-α, IL-6, and IFN-γ in the NAFLD group compared to the healthy group. These factors, along with the related impairment in mucosal immune function, play a significant role in the pathogenesis of NAFLD.	(32)
Melanoma	Patients with melanoma and healthy volunteers.	Patients diagnosed with melanoma exhibited a higher relative abundance of *Fusobacterium* compared to the control group. In early-stage melanoma, there was an increased alpha diversity and a higher abundance of the genus *Roseburia* compared to the control group.	Inflamation: Regulating the immune system.	(33)
LC	Mouse model and cell lines.	Several bacterial taxa, including *Herbaspirillum* and *Sphingomonadaceae*, were notably over-represented in tumor-bearing lungs. A variety of other taxa, such as *Aggregatibacter* and *Lactobacillus*, were found to be enriched in healthy lungs.	Inflammation: Inflammation associated with lung adenocarcinoma by activating γδ T cells that reside in the lungs. Symbiotic bacteria stimulate myD88-dependent IL-1B and IL-23 production in bone marrow cells, induce proliferation and activation of Vg6 + Vd1 + γδ T cells, mediate inflammation by inducing production of effector molecules such as IL-17, and lead to tumor cell proliferation in LC.	(34)
LC	LC patients and healthy controls.	The genus *Streptococcus* showed a significantly higher abundance in cancer cases compared to the controls, whereas *Staphylococcus* was more abundant in the control group. There was an increasing trend in the abundance of the genera *Streptococcus* and *Neisseria*, while *Staphylococcus* and *Dialister* exhibited a gradual decline from healthy to noncancerous to cancerous sites.	Inflammation: Microbiota-mediated inflammation	(35)
LC	Patients referred with possible LC.	Among the seven bacterial species present in all samples, *Streptococcus viridans* exhibited a significantly higher abundance in LC+ samples. Seven bacterial species were exclusive to LC-, while 16 were unique to samples from LC+ individuals. The abundance of Granulicatella adiacens showed a correlation with six other bacterial species (*Enterococcus sp. 130, Streptococcus intermedius, Escherichia coli, S. viridans, Acinetobacter junii*, and *Streptococcus sp. 6*) in LC+ samples only.	Metabolic reprogramming: Functional differences, as indicated by significant fold changes, included alterations in polyamine metabolism and iron siderophore receptors.	(36)
LC	Patients who had undergone bronchoscopies.	The relative abundance of two phyla, *Firmicutes* and *TM7*, was significantly increased in patients with LC. Two genera, *Veillonella* and *Megasphaera*, were found to be relatively more abundant in LC patients.	Inflammation: Microbiota-mediated inflammation	(37)
CC	Four groups of women (cervical cancer, HPV-positive CIN, HPV-positive non-CIN, and HPV-negative groups).	In the cervical cancer group, the abundance of *Lactobacillus* decreased, while the abundance of *Prevotella* and *Gardnerella* increased.	Inflamation: Dyobisis of vaginal microbiota contributes to the disruption of immune function, leading to an increase in immune inflammatory factors (IP-10 and VEGF-A). Consequently, this creates a favorable inflammatory environment conducive to the occurrence of cancer.	(38)
LC	LC patients and healthy individuals.	Individuals exhibiting decreased alpha diversity were observed to have an elevated risk of LC. The presence of *Fusobacterium nucleatum* was identified in association with LC risk.	Inflammation: Microbiota-mediated inflammation	(11)
SCC	Mouse model and chronic periodontitis patients.	The oral microbiota associated with periodontitis maintained a dominant position throughout the entire process of OSCC with periodontitis, with *Porphyromonas* being the most abundant genus.	Inflammation: The oral microbiota linked to periodontitis was found to directly activate interleukin-17-positive (IL-17+) γδ T cells. These activated γδ T cells played a crucial role in activating the IL-17/signal transducer and activator of transcription 3 (STAT3) pathway, and promoting the infiltration of M2-tumor-associated macrophages (TAMs) in OSCC proliferation.	(39)
CRC	CRC patients and controls.	Within the phylum *Actinobacteria, Bifidobacteriaceae* exhibited higher abundance among CRC patients compared to controls. In the phylum *Bacteroidetes, Prevotella denticola* and *Prevotella sp.* oral taxon 300 were identified to be associated with an increased risk of CRC.	Inflammation: Microbiota-mediated inflammation	(40)
CRC	CRC patients.	More than 40% of CRC patients displayed identical strains of *Fusobacterium nucleatum* in both their CRC tissue specimens and saliva samples.	Inflammation: Microbiota-mediated inflammation	(41)
EC	EAC and ESCC patients and controls.	The presence of the periodontal pathogen *Tannerella forsythia* was associated with a higher risk of EAC. The abundance of the periodontal pathogen *Porphyromonas gingivalis* showed a trend towards a higher risk of ESCC.	Inflammation: Microbiota-mediated inflammation	(42)
PaC	PaC patients and controls.	The carriage of oral pathogens, specifically *Porphyromonas gingivalis* and *Aggregatibacter actinomycetemcomitans*, was linked to a higher risk of pancreatic cancer.	Inflammation: Microbiota-mediated inflammation	(43)
SCC	SCC, AK patients, and healthy controls.	In SCC, the relative abundance of the pathobiont *Staphylococcus aureus* was increased, while the commensal *Cutibacterium acnes* was decreased compared to healthy skin. The association of *Cutibacterium acnes* with lesional versus healthy skin differed at the strain level.	Inflammation: Microbiota-mediated inflammation	(44)
BC	Breast tumor and cell lines.	At the genus level, the proportional abundance of *Brevunimonas* and *Staphylococcus* was increased in patients with primary breast tumors who later developed metastatic disease.	Genome instability Induce DNA double-stranded breaks	(45)
Melanoma	Piglets.	*Lactobacillus, Clostridium sensu stricto 1*, and *Corynebacterium 1* were among the most discriminately higher genera in the healthy skin microbiome, whereas *Fusobacterium, Trueperella*, *Staphylococcus, Streptococcus*, and *Bacteroides* were discriminately abundant in melanoma tissue microbiome. In the faecal microbiota of MeLiM piglets, *Bacteroides, Fusobacterium*, and *Escherichia-Shigella* were found to be associated.	Metabolic reprogramming: Significant differences were observed in the predicted metabolic profiles between the healthy skin microbiome and melanoma tissue microbiome. The faecal microbiome of MeLiM piglets exhibited enrichment in genes related to membrane transport pathways, potentially contributing to increased intestinal permeability and alterations in the intestinal mucosal barrier.	(46)
LC	LUAD and LUSC patients.	There were significant differences in gene expression and microbial abundance associated with recurrence and metastasis between LUAD and LUSC. In LUSC, the bacterial community associated with recurrence and metastasis (RM) exhibited lower richness compared to non-RM cases. There were significant correlations between host genes and tissue microbes in LUSC, while such host-tissue microbe interactions were rare in LUAD.	Metabolic reprogramming: A set of pathways was identified that showed an association with specific tissue microbiome composition in LUSC. These pathways primarily involved various metabolic and metabolism-related enzymes, some of which have been previously implicated in LC, including drug metabolism-cytochrome P450, metabolism of xenobiotics by cytochrome P450, and steroid hormone biosynthesis.	(12)
PaC	Mouse model and cell lines.	Tumor microbiome was abundant in anaerobic *Bacteroidales* in hypoxic and immunosuppressive tumors.	Sustaining proliferative signaling: The homotrimeric form of Collagen Type 1 (Col1 α1/α1/α1) derived from pancreatic cancer cells has been demonstrated to facilitate oncogenic signaling via DDR1 and integrin α3β1. This process results in an increased abundance of *Bacteroidales* within the intratumoral microbiome.	(47)
CRC	Tissue from the tumors of CRC patients.	In CRC tumors, *Fusobacterium* and *Bacteroides* emerge as the most dominant genera.	Invasion and metastasis: CRC cells infected with *Fusobacterium nucleatum* showcase heightened invasiveness into their surrounding environment. These infected cells attract myeloid cells to the bacterial niches, accelerating migration rates significantly. This process is mediated through various signaling pathways crucial for cancer metastasis, including extracellular matrix remodeling and modulation of cell adhesion and migration via ERK1 and ERK2.	(48)
CRC	CRC cell lines.	CRC cell lines infected with *Fusobacterium nucleatum* formed larger tumors, more rapidly in nude mice compared to uninfected cells.	Inflammation: Several inflammatory factors, including interleukin 17F, interleukin 21, interleukin 22, and MIP3A, were significantly increased in the serum of mice given *Fusobacterium nucleatum.*Invasion and metastasis: *Fusobacterium nucleatum* activates Toll-like receptor 4 signaling to MYD88, leading to activation of the nuclear factor-κB and increased expression of miR21; this miRNA reduces levels of the RAS GTPase RASA1. Patients with both high amount of tissue *F.nucleatum* DNA and miR21 demonstrated a higher risk for poor outcomes.	(49)
CRC, GC	CRC and GC patients.	In CRC, *Fusobacterium, Bacteroides*, and *Ruminococcus* were found to be highly enriched. In GC, *Streptococcus, Acinetobacter*, and *Brevundimonas* dominated.	Genome instability: DNA repair-associated microbiota were observed in CRC, including mismatch repair, DNA repair, and recombination proteins and DNA replication proteins. Metabolic reprogramming: The microbiotas in GC were associated with central carbon and amino acid metabolism pathways, such as glyoxylate and dicarboxylate metabolism, and glycine/serine/ threonine metabolism.	(50)
GC	GC patients.	The abundance of *Helicobacter* was observed to be increased in non-tumor tissues, while the abundance of *Lactobacillus, Streptococcus, Bacteroides, Prevotella*, and six additional genera was increased in tumor tissues.	Metabolic reprogramming: The differences in metabolome profiles between GC tumor and matched non-tumor tissues may be attributed, in part, to the collective activities of *Helicobacter, Lactobacillus*, and other bacteria. These activities are believed to influence GC carcinogenesis and progression.	(51)
BC	Mouse model.	The direct administration of specific bacterial strains, including *Staphylococcus* and *Lactobacillus*, isolated from the microbiota of breast tumors, was shown to promote metastasis in experimental tumor models.	Invasion and metastasis: During metastatic colonization, intratumor bacteria carried by circulating tumor cells played a role in promoting host- cell survival. This was achieved by enhancing resistance to fluid shear stress through the reorganization of the actin cytoskeleton.	(52)
BC	BC tissues and breast control samples from healthy individuals.	In all four types of BC (ER positive, triple positive, Her2 positive, and triple-negative BCs), dominant microbial signatures were observed for *Proteobacteria*, followed by *Firmicutes*. *Actinomyces* signatures were detected in each of these BC types.	Metabolic reprogramming: Impact estrogen metabolism Inflammation: Microbiota-mediated inflammation	(53)
BC	Fresh breast tissue was collected from women undergoing breast surgery.	The breast tissue of women with BC exhibited higher relative abundances of *Bacillus, Enterobacteriaceae, Staphylococcus, Escherichia coli* (a member of the *Enterobacteriaceae* family), and *Staphylococcus epidermidis* compared to healthy women.	Genome instability Induce DNA double-stranded breaks	(54)
Multiple types	Tumors (seven cancer types) and their adjacent normal tissues.	Colorectal tumors exhibited *Firmicutes* and *Bacteroidetes* phyla as the most abundant species. The microbiome of pancreatic cancer was characterized by the dominance of *Proteobacteria*, akin to the normal duodenal microbiome. Across various cancer types, species from the *Proteobacteria* and *Firmicutes* phyla were predominant in the detected bacterial sequences. However, the *Proteobacteria* to *Firmicutes* (P/F) ratio varied among different tumor types. Taxa from the *Actinobacteria* phylum, particularly the *Corynebacteriaceae* and *Micrococcaceae* families, were prevalent in nongastrointestinal tumors like breast, lung, and ovarian cancer. *Fusobacterium nucleatum*, previously linked to enrichment in colorectal tumors, was also identified in breast and pancreatic tumor cohorts.	Metabolic reprogramming: The unsupervised clustering analysis of 287 predicted metabolic MetaCyc pathways, which exhibited the greatest variability between tumor types, revealed that specific microbiome metabolic pathways were relatively specific to certain tumor types.	(55)

AK, actinic keratosis; BC, Breast cancer; Bft, Bacteroides fragilis toxin; CC, Cervical cancer; CRC, Colorectal cancer; EAC, Esophageal adenocarcinoma; EC, Esophageal Cancer; EO-CRC, Early-onset CRC; ER, Endocrine receptor; ESCC, Esophageal squamous cell carcinoma; GC, Gastrointestinal cancer; IL-6, Interleukin-6; LC, Lung cancer; LiC, Liver cancer; LO-CRC, late-onset CRC; LUAD, Lung adenocarcinoma; LUSC, Lung squamous cell carcinoma; MAMP, Microorganism-associated molecular patterns; NLR, Neutrophil-to-lymphocyte ratio; OSCC, Oral squamous cell carcinoma; OTUs, Operational taxonomic units; PaC, Pancreatobiliary cancer; PLR, Platelet-to-lymphocyte ratio; PRRs, Pattern recognition receptors; PC, Prostate Cancer; RM, Recurrence or metastasis; sCTLA-4, Soluble cytotoxic T lymphocyte associated antigen-4.

Numerous studies have revealed that tumors can interact with various components within the body as well as metabolisms (57), such as platelets (58), circulating tumor cells (CTCs) (59), exosomes (60), and modify these components to serve the tumor’s growth. Similarly, an important mechanism explaining the causal relationship between the human microbiome and cancer development is the interplay between microbiota and tumor cells, leading to significant alterations in the composition and function of microbiomes in cancer patients. The dysbiosis in various microbiota has been observed in cancer patients with changed variability of ecosystems compared to healthy people. *Fusobacterium nucleatum* and *Parvimonas micra* were found to be more prevalent, while *Clostridia* and *Bacteroidia* decreased in the gut of colorectal cancer (CRC) patients (61). Additionally, *Lactococcus* and *Fusobacteria* exhibited higher abundance, whereas *Pseudomonas* and *Escherichia-Shigella* were downregulated in CRC tissues compared to adjacent non-cancerous ones (62, 63). Likewise, the gut microbiota of cervical cancer patients exhibited notable variations in the abundance of seven genera, namely *Escherichia-Shigella*, *Roseburia*, *Succinivibrio*, *Lachnoclostridium*, *Lachnospiraceae_UCG-004*, *Dorea*, and *Pseudomonas* (30).

Reprogrammed ecosystems were also identified in cancer patients beyond the gastrointestinal tract, involving other different microbiota. Cervical cancer exhibits the greatest diversity in vaginal microbiota, with the enrichment of *Ralstonia,Lactobacillus, Gardnerella, Sneathia and Prevotella*. Similarly, *Gardnerella*, *Prevotella*, and *Sneathia* exhibit higher prevalence within the HPV-positive cervical intraepithelial neoplasia (CIN) group. In contrast, *Gardnerella* and *Prevotella* are more prevalent in the HPV-positive non-CIN group (38).

The significance of microbiota variation in tumorigenesis is evident when comparing the microbiome across healthy tissue, pre-cancerous lesions, and malignant tissues. A transitional microbial dysbiosis is observed from healthy skin to pre-malignant actinic keratosis (AK) and further to squamous cell carcinoma (SCC), marked by an elevated presence of the pathobiont *Staphylococcus aureus*, surpassing the commensal *Cutibacterium acnes* in SCC (44). In a recent study, *Bacteroides*, *Trueperella*, *Staphylococcus*, *Streptococcus*, and *Fusobacterium* were found to be notably more abundant in the microbiome of melanoma tissue, while *Corynebacterium* 1, *Clostridium* sensu stricto 1, and *Lactobacillus* were identified as the genera exhibiting significantly higher levels in the microbiome of healthy skin (46). Furthermore, there is a significant differentiation in the microbial enrichment of microbiome between patients at various stages of cancer (33). The well-documented heterogeneity of tumor cells and the tumor microenvironment extends beyond diversity at the cellular and molecular levels to include microbial clusters forming micro niches with varying species composition and quantities within a tumor mass. This specific distribution of clusters has been reported with multiple types of tumors, from skin cancer to CRC or gastric cancer (48, 50).

### 2.2 Reprogrammed microbiota impact cancer hallmarks

Not only limited to changes in microbial composition, in the cancer state, there are significant alterations in the functions of microorganisms, especially in metabolism, generating metabolites that favor the hallmarks of cancer cells (**Table 1**). The toxin produced by enterotoxigenic *Bacteroides fragilis* (ETBF), known as *B. fragilis* toxin (BFT), is implicated in colitis and prompts a pro-cancerous inflammatory response. This inflammation, driven by Stat3 and T helper type 17 (T(H)17) cells, contributes to colonic hyperplasia and the development of tumors (64). Similarly, through the Stat3 pathway and interleukin-17-positive (IL-17+) γδ T-cells axis, the oral microbiota found in periodontitis, particularly with a dominant presence of *Porphyromonas*, has been implicated in the promotion of oral SCC (39). The homotrimeric form of Collagen Type 1, comprising three α1 chains, derived from pancreatic cancer cells, has been shown to facilitate oncogenic signaling via Discoidin domain receptor 1 and integrin α3β1. This signaling pathway promotes cancer cell proliferation and the formation of tumor organoids. Additionally, it leads to an increased abundance of *Bacteroidales* within the intratumoral microbiome (47).

Angiogenesis is another important hallmark, essentially contributing to the growth of tumors. There is ample evidence demonstrating the relationship between microbiomes at various locations in the body, from the gut to ocular microbiota, and the process of neovascularization (65, 66). The immortality of tumor cells is originated from the capability to resist apoptosis. On the other hand, apoptosis can be impacted by metabolites from certain taxa in various microbiota. *P. anaerobius*, found in abundance in CRC patients, secretes trans-3-indoleacrylic acid (IDA), which promotes CRC development by counteracting ferroptosis, a form of cell death characterized by uncontrolled lipid peroxidation and subsequent membrane damage. Inhibiting key mediators of IDA, such as Apoptosis-inducing factor 2, aldehyde dehydrogenase 1 family member A3, or Aryl Hydrocarbon Receptor, reversed this effect and suppressed tumor growth. On the other hand, feeding IDA or introducing *P. anaerobius* accelerated CRC development in mouse models (67). In recent years, advancements in next-generation sequencing (NGS) technology have facilitated the exploration of interactions between metagenomics across diverse microbiomes and the host genome. In a case-control study, a correlation was discovered between the colon microbiota and specific mutations and genome stability in CRC tumors. This study revealed that the presence of the Kirsten rat sarcoma virus (KRAS) mutant type was positively linked to *Faecalibacterium* while inversely associated with the presence of *Bifidobacterium* (18).

A characteristic of cancer cells leading to recurrence and treatment failure is their ability to metastasize and invade surrounding tissues. The role of gut microbiota in the dissemination, survival, and colonization of metastatic cancer cells has long been recognized through numerous studies across various cancer types (68). Recently, along with the discovery of microbiota residing within tumors, their significant role in tumor migration and metastasis has been unveiled. Research conducted on a murine model of spontaneous breast tumors revealed that during the process of metastatic colonization, bacteria residing within the tumor were transported by CTCs. These bacteria played a role in enhancing the survival of host cells by increasing their resistance to fluid shear stress during the metastasizing process through the reorganization of the actin cytoskeleton. Additionally, the direct administration of certain bacterial strains, such as *Staphylococcus* and *Lactobacillus*, isolated from the microbiota of breast tumors, promoted metastasis in experimental tumor models. Conversely, when breast intratumor bacteria were depleted, there was a significant reduction in lung metastasis (52). CRC cells infected with *F. nucleatum* exhibit enhanced invasiveness into their surrounding environment and attract myeloid cells to the bacterial niches. This process accelerates migration rates significantly by mediating various signaling pathways crucial for cancer metastasis, including extracellular matrix remodeling and modulation of cell adhesion and migration via ERK1 and ERK2 (48).

### 2.3 Reprogrammed microbiota modifies the host immune system

The characteristic changes in microbiota composition in cancer patients can result in alterations of the composition and functions of immune cells including T-cells, natural killer (NK) cells, dendritic cells (DCs), and macrophages, implicated in antitumor immunity. A comparative analysis of intraepithelial lymphocytes in CRC tissue versus healthy colonic tissue revealed a reduction in γδ T-cells and resident memory T-cells within cancerous tissue. These populations exhibited a regulatory CD39-expressing phenotype in the cancer microenvironment. Moreover, distinct patterns of T-cell proliferative responses to various commensal bacteria were observed in CRC patients, while B cell memory responses to certain bacteria/yeast were notably elevated. This increase in B cell memory responses was accompanied by higher proportions of circulating effector memory B cells, transitional B cells, and plasmablasts (69).

The influence of microbiota on lymphocyte populations is partially attributed to the modulation of antigen presentation cells through microbiota metabolites. Specifically, butyrate, a short-chain fatty acid (SCFA) metabolite produced by microbiota, has been shown to hinder DCs presentation of tumor-associated antigens. Consequently, this impediment affects the infiltration of T-CD8+ cells in an IFN-γ-dependent manner. Therefore, the depletion of butyrate-producing strains in the gut microbiota through vancomycin treatment has been observed to enhance the antitumor response to radiotherapy (70). Likewise, phytosphingosine, a metabolite derived from the gut microbiota, can increase the expression of HLA class I on cancer cells. This sensitizes the cells to targeted antigen-specific cytotoxic T lymphocyte destruction, both *in vitro* and within living organisms, thereby enhancing the efficacy of immuno checkpoint inhibitor (ICI) treatments (71).

Besides altering the antigen-presenting function of immune cells, microbiota also has the capability to influence the production of pro-inflammatory cytokines like interleukin-12 (IL-12) and IFN-γ. These cytokines play crucial roles in activating and enhancing the function of cytotoxic T-cells and NK cells (72, 73). Apart from the systemic immune modulation exerted by gut microbiota, the intratumoral microbiota plays a role in shaping the immune profile within the tumor microenvironment. In oral SCC, an enrichment of genera such as *Capnocytophaga*, *Fusobacterium*, and *Treponema* correlated with the presence of effector subsets of tumor-infiltrating lymphocytes and the associated gene expression involved in the recruitment of B cells and T-cells. This enrichment ultimately leads to immunosuppressive effects within the tumor microenvironment (74).

Fascinatingly, the microbiota not only can interact with tumors to modulate immune responses toward decreased tumor surveillance, but it can also interact with therapeutic drugs to alter the immune system in a synergistic antitumor direction. A recent study revealed that ICI induces the movement of specific native gut bacteria into secondary lymphoid organs and subcutaneous melanoma tumors. Specifically, ICI prompts the restructuring of lymph nodes and activation of DCs, facilitating the migration of a specific subgroup of gut bacteria to extraintestinal tissues. This migration promotes optimal antitumor T-cell responses in both the tumor-draining lymph nodes (TDLNs) and the primary tumor. Furthermore, antibiotic treatment leads to reduced translocation of gut microbiota into mesenteric lymph nodes (MLNs) and TDLNs, resulting in weakened DCs and effector CD8+ T-cell responses, as well as diminished responses to ICI. These findings offer opportunities for leveraging microbiota in a beneficial direction for treatment (75).

Immune checkpoint molecules, such as programmed cell death protein 1 (PD-1), Programmed Death Ligand 1 (PD-L1), and cytotoxic T-lymphocyte-associated protein 4 (CTLA-4), play pivotal roles in regulating T-cell responses. Research indicates that certain microbial species and their metabolites can influence the expression of these checkpoint molecules, thereby impacting T-cell activation and facilitating immune evasion by tumors. A study examining the urogenital microbial community in bladder cancer patients revealed an increased presence of *Leptotrichia*, *Roseomonas*, and *Propionibacterium*, along with a reduction in certain bacterial genera, such as *Prevotella* and *Massilia*, among those exhibiting PD-L1 expression on cancerous tissues compared to those who tested negative for PD-L1 expression (76). Similarly, *Veillonella dispar* was prevalent in the lung microbiome of lung cancer patients exhibiting high PD-L1 expression, whereas the abundance of *Neisseria* was notably elevated in patients with low PD-L1 expression. Consequently, *V. dispar* predominated in the group of patients showing a positive response, whereas *Haemophilus influenzae* and *Neisseria perflava* were prevalent in the non-responder group (77). In the context of CTLA4 blockade therapy, heightened concentrations of butyrate and propionate in the bloodstream correlate with treatment resistance and an increased proportion of Treg cells. Mouse studies reveal that butyrate impeded the CTLA-4-induced elevation of CD80/CD86 expression on DCs and ICOS expression on T-cells, along with the buildup of tumor-specific T-cells and memory T-cells. In patients, elevated blood butyrate levels mitigated the ipilimumab-induced accumulation of memory and ICOS + CD4 + T-cells and IL-2 production, suggesting that SCFA restricts the efficacy of anti-CTLA-4 therapies (78).

Overall, the molecular intricacies governing the interplay between the microbiome and antitumor immunity encompass complex interactions among microbial elements, host immune cells, and the tumor microenvironment. Grasping these mechanisms is imperative for devising microbiome-centered interventions aimed at bolstering antitumor immune responses and refining cancer treatment outcomes.

## 3 The impact of microbiota on cancer treatment

**Figure 1:**
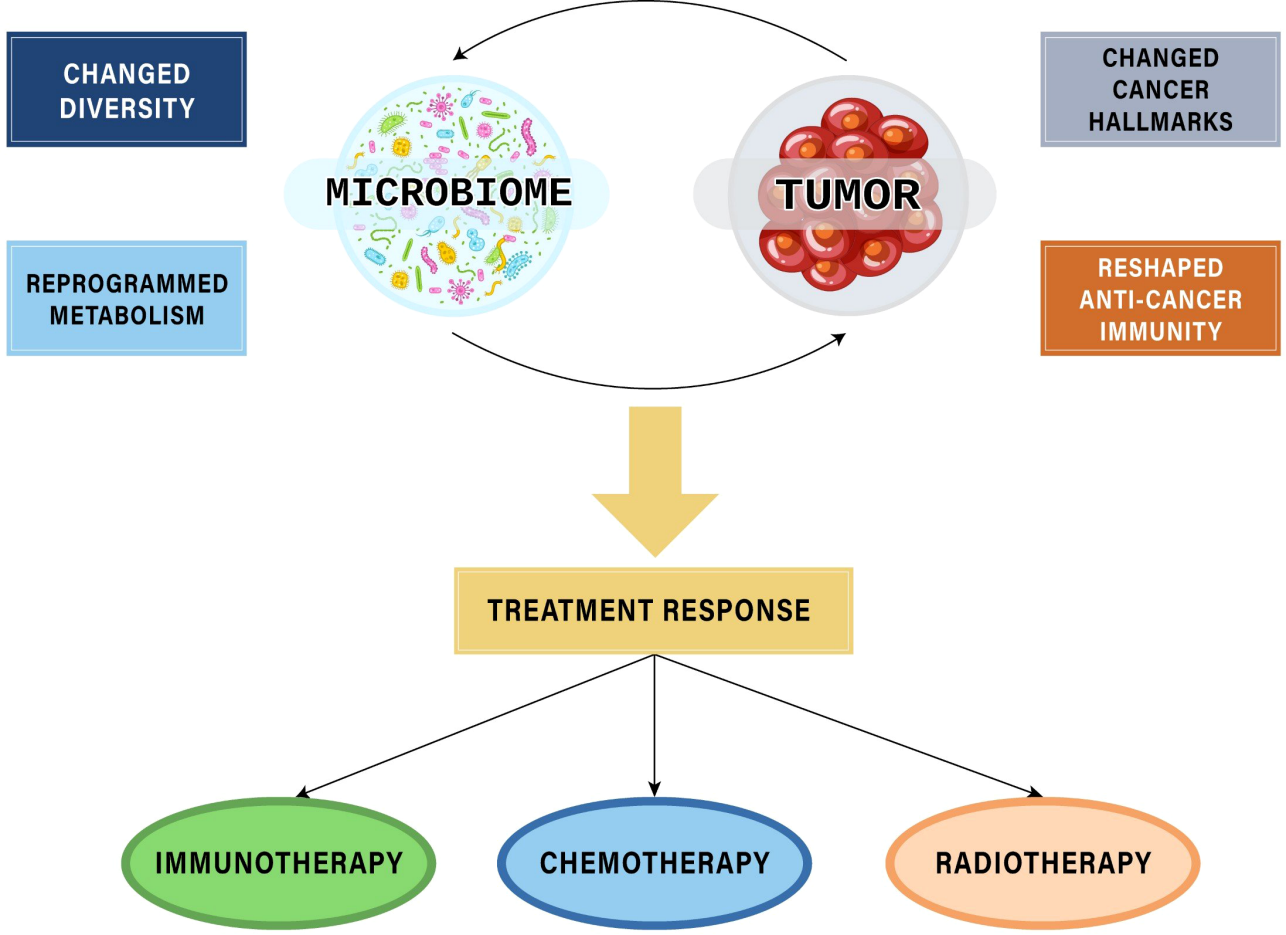
The interplays between microbiomes and tumors affecting responses to cancer treatment modalities.

Given the crucial role in tumor formation, development, metastasis, and host immunity, microbiota can exert significant influences on patients’ responses to cancer treatment modalities, including both therapeutic efficacy and toxicity. Among these therapies, the most extensively investigated area with compelling evidence supporting the role of microbiota is the field of immunotherapy - the latest cancer treatment method that has made remarkable advances in clinical application. Additionally, microbiota also affect response to other cancer therapies, suggesting perspectives of interventions to achieve precision medicines (**Figure 1**).

### 3.1 Microbiota and immunotherapy response

In precision medicine, the most important aspect is to identify biomarkers for the stratification of patient groups to select appropriate drugs for each group. Although some molecular biomarkers have been applied in personalized medicine with immunotherapies, their true effectiveness in clinical practice remains controversial, requiring supplementation or support from other types of biomarkers. Significant variations in the composition of gut microbiota have been observed between patients who respond favorably to ICI and non-responders across a range of different cancer types. Responding melanoma patients exhibited notably higher alpha diversity, along with a relative abundance of *Ruminococcaceae* bacteria. Intriguingly, metagenomic analysis revealed an enrichment of amino acid biosynthesis in responders, thereby contributing to enhanced immunity characterized by increased infiltration of CD4+ and CD8+ T-cells (79). Likewise, an analysis of gut microbiota utilizing 16S ribosomal RNA sequencing revealed increased alpha diversity among responders to ICI and CTLA-4 inhibitors across various cancer types. The microbiota composition of responders resembled that of healthy individuals. Additionally, certain bacteria, including *Prevotella copri* and *Faecalibacterium prausnitzii*, were linked to a favorable treatment outcome (80). Together with the enhanced alpha diversity, the enrichment of g-Blautia has been suggested as a potential predictor of responders to ICI with longer progression-free survival (PFS) in patients with non-small cell lung cancer (NSCLC) (81). Interestingly, Sarfaty et al. not only identified cancer type-specific microbiome signatures to distinguish between favorable responders and non-responders but also observed certain similarities in the microbiome signatures of non-responders across three different cancer types including lung, urothelial, and melanoma. This suggests the potential utility of these signatures as common pharmacomicrobiomic biomarkers (82).

Despite the treatment efficacy advantages of such an advanced therapeutic modality, ICI, like other cancer treatment regimens, also have unintended effects, notably immune-related adverse events (irAEs), which can impact treatment response and patient adherence. Among melanoma patients, responders experiencing irAEs from grade 2 in the Common Terminology Criteria for Adverse Events (CTCAE) exhibited a predominance of *Bacteroides plebeius* and *Bacteroides coprophilus* in their gut microbiota, while those without irAEs showed an enrichment of *Eubacterium siraeum* (83). Apart from alterations in gut microbiota, there is also a distinction in the skin microbiome among melanoma patients experiencing cutaneous irAEs following ICI treatment. This is characterized by an increase in *Staphylococci* and *Proteobacteria*, whereas patients without irAEs exhibited enrichment in anaerobic *Eubacteriales* (84). Identifying predictors of irAEs can aid in mitigating severe ADRs and preventing patient suffering.

Beyond merely identifying differences in microbiota between patient groups responding and not responding to immunotherapies, scientists can actively modify treatment responses by intervening in the microbiota. *In vivo* experiments have shown that transplanting fecal material from patients who respond to treatment with an abundance of *Bifidobacterium longum*, *Collinsella aerofaciens*, and *Enterococcus faecium* into germ-free mice could result in enhanced tumor control, increased T-cell responses, and improved efficacy of anti-PD-L1 therapy (85). Conversely, mice that received fecal transplants from patients with poor responsiveness exhibited elevated frequencies of regulatory CD4+FoxP3+ T-cells and CD4+IL-17+ T-cells in the spleen. This indicates compromised host immune responses, ultimately resulting in the failure of ICI treatment (79). A thorough study on improving the gut microbiota to finetune cancer immunotherapy shows that certain bacteria in the gut are very important in determining the immune responses linked to CTLA-4 checkpoint blocking therapy. By regulating the gut flora, this understanding has the potential to enhance the therapeutic effectiveness of ICI and potentially reduce its immune-mediated toxicity (86).

In addition to impacting gut microbiota through fecal transplantation, skin bacteria can be genetically modified to induce changes in systemic anti-cancer immune responses. A recent study revealed that modifying the skin bacterium *Staphylococcus epidermidis* to express tumor antigens could enhance highly specific adaptive immune responses mediated by T-cells, leading to significant improvements in melanoma immunotherapy efficacy (87). Additionally, a more straightforward approach to modulate gut microbiota and thus modify the response to ICI treatment is through dietary interventions. Melanoma patients who consumed a diet rich in dietary fiber experienced significantly extended PFS while undergoing ICI treatment. Conversely, mice fed a low-fiber diet exhibited poor responsiveness to anti-PD1 therapy, characterized by a reduced frequency of interferon-γ–positive cytotoxic T-cells in the tumor microenvironment (88).

### 3.2 Microbiota and chemotherapy response

**Figure 2:**
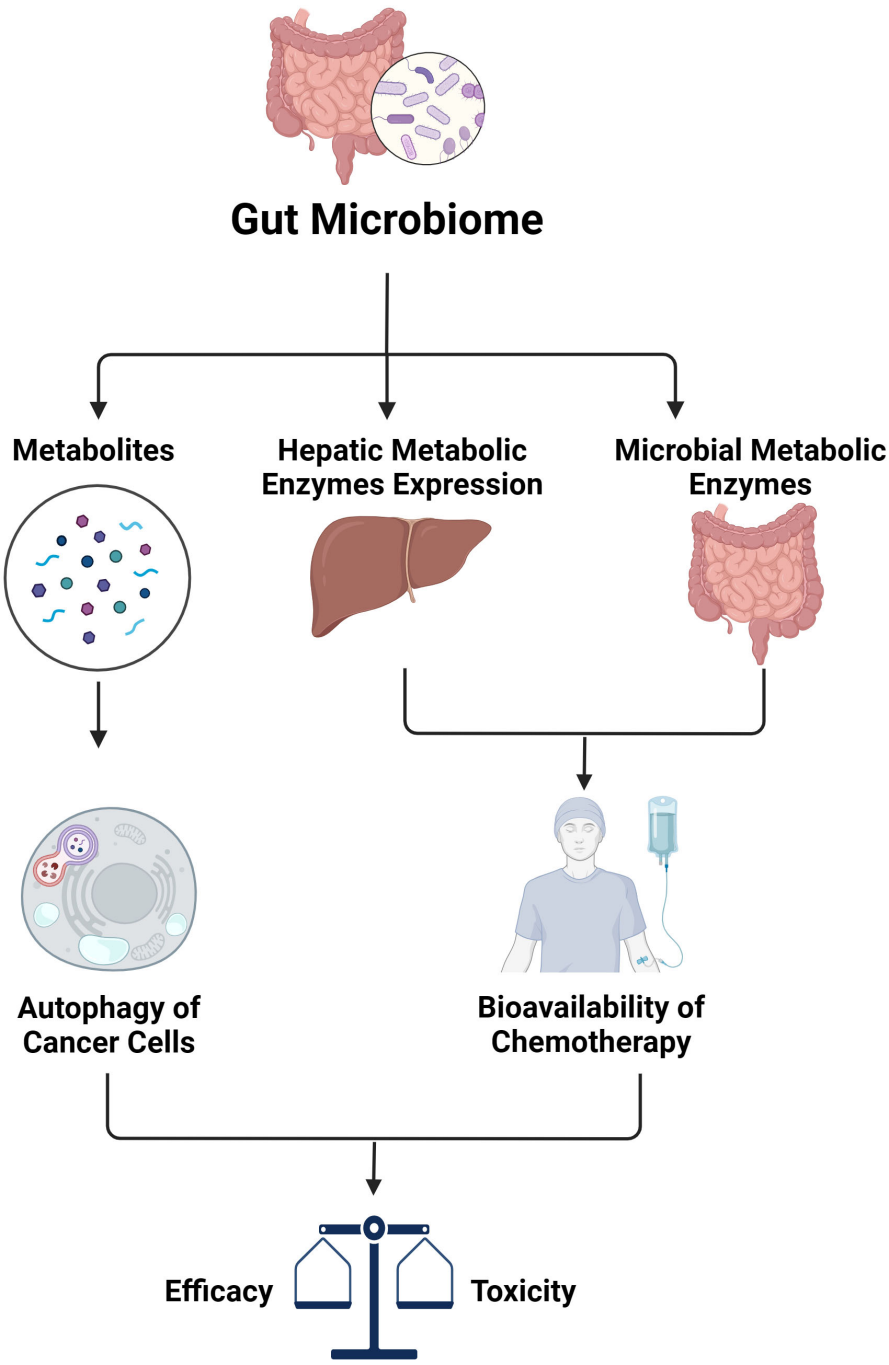
The impact of gut microbiome on efficacy and toxicity of chemotherapy (created with BioRender.com).

In addition to its impact on immunotherapy, the microbiota plays a significant role in influencing the effectiveness of other treatments such as chemotherapy, whose response relies heavily on pharmacokinetics and pharmacodynamics (**Figure 2**). Like many other therapeutic groups, the bioavailability of chemotherapy agents is determined by hepatic metabolic enzymes and transporters, including the cytochrome P450 superfamily, which is responsible for metabolizing a majority of medications. Research has shown that alterations in gut microbiota can affect the expression of key pharmacokinetic proteins like CYP3A1, UGT1A1, and P-glycoprotein (P-gp) in the liver. Specifically, changes in the composition of gut microbiota have been observed to impact the metabolism and bioavailability of drugs like cyclosporine. For instance, higher levels of *Alloprevolleta* and *Oscillospiraceae* UCG 005 have been associated with reduced bioavailability of cyclosporin, while increased levels of *Parasutterella* and the *Eubacterium xylanophilum* group have been linked to increased bioavailability (89). Previously, only the biotransformation processes of drugs by human hepatic enzymes were known. However, recent studies have shown that enzymes from the intestinal microbiota also participate in drug metabolism reactions, significantly affecting the plasma concentration of drugs and their metabolite as well as the drug’s elimination half-life (90, 91). Consequently, this impacts the efficacy and toxicity of the treatment. This is particularly significant for drugs with a narrow therapeutic window such as chemotherapies.

Similar to immunotherapies, there exists a significant disparity in both the composition and abundance of microorganisms within the gut microbiota between patients who respond favorably to chemotherapy and those who do not, across various types of cancer. This contrast has been observed in locally advanced rectal cancer patients, where intestinal microbes associated with butyrate production, such as *Roseburia*, *Dorea*, and *Anaerostipes*, were found to be more prevalent in responders to neoadjuvant chemotherapy, whereas *Coriobacteriaceae* and *Fusobacterium* were more prevalent in non-responders. A set of ten predictors, including *Dorea*, *Anaerostipes*, and *Streptococcus*, was identified for the stratification of responders, achieving an area under the curve value of up to 93.57% (92).

The favorable efficacy of chemotherapies is associated with some specific microbiota-derived metabolites, suggesting their utility as solutions to enhance the benefit of such a popular cancer therapy. The microbiota-derived tryptophan metabolite, indole-3-acetic acid (3-IAA) which plays a crucial role in the autophagy process of cancer cells was found in higher concentrations in pancreatic cancer patients who positively responded to treatment. Studies have shown that interventions such as fecal microbiota transplantation, short-term dietary adjustments focusing on tryptophan, and oral administration of 3-IAA enhance the effectiveness of chemotherapy in humanized murine models of pancreatic cancer. Furthermore, a significant correlation between the levels of 3-IAA and the chemotherapy’s efficacy has been observed in two separate cohorts of patients with pancreatic ductal adenocarcinoma (PDAC) (93). By utilizing machine learning models incorporating both drug response and microbiota data, Hermida et al. demonstrated that the microbiota profile emerges as a superior predictor of clinical outcomes when compared to clinical variables across seven distinct cancer types, including chemotherapy treatments for bladder urothelial carcinoma, docetaxel treatment for breast invasive carcinoma and sarcoma, as well as various treatments for stomach adenocarcinoma (94).

Not only can the microbiota profile predict the efficacy of chemotherapies, but it can also help anticipate specific ADRs caused by chemotherapy agents. In acute lymphoblastic leukemia (ALL), the initial gut microbiome composition, marked by an abundance of *Proteobacteria*, served as a predictive factor for febrile neutropenia following chemotherapy. Notably, a prevalence of *Enterococcaceae* was associated with a significantly higher likelihood of experiencing subsequent febrile neutropenia and diarrheal ADRs. Additionally, the dominance of *Streptococcaceae* predicted a remarkably increased risk of subsequent diarrheal adverse events (95). Similarly, the presence of *Bacteroides* and *Blautia2* in the gut microbiota of rectal cancer patients could predict ADRs such as fatigue, sleep disturbance, or depression following chemotherapy with an accuracy of 74% (96). Also in rectal cancer cases, dynamic changes observed in the tumor microbiome throughout and following chemoradiation therapy were associated with drug-related toxicity. Specifically, patients who experienced heightened toxicity by week 5 displayed elevated relative counts of *Clostridia*, *Actinobacteria*, and *Clostridiales* at the outset of treatment (97).

### 3.3 Microbiota and radiotherapy response

Radiotherapy stands as a crucial cancer treatment modality with interindividual variations in therapeutic effectiveness and toxicity. Among its most significant antitumor mechanisms is the stimulation of both innate and adaptive immunity. Consequently, the microbiota, which exerts profound influences on the host immune system, can significantly influence therapeutic outcomes. The intricate relationship between intestinal microbiota and post-radiation immune responses in mouse models of breast cancer and melanoma has been uncovered. While the exclusion of gut fungi enhanced the anti-tumor effects of radiation, the use of antibiotics to deplete bacteria diminished responsiveness, leading to the proliferation of commensal fungi. Moreover, the expression level of intratumoral Dectin-1, a key innate fungal sensor was essential for the impact of commensal fungi in mice undergoing radiation therapy and could predict survival rates in breast cancer (98).

Butyrate, a common metabolite produced by intestinal bacteria, is renowned for its impact on immune function. Recent research using murine models has revealed a negative correlation between the abundance of butyrate-producing gut bacteria and anticancer responses to radiation. Butyrate inhibited STING-activated type I IFN expression in DCs by blocking TBK1 and IRF3 phosphorylation. This inhibition abolished radiation-induced tumor-specific cytotoxic T-cell immune responses, without directly shielding CRC and melanoma cells from radiation. These results underscore the potential of selectively targeting butyrate-producing microbiota as a novel therapeutic approach to enhance tumor radiation sensitivity (99). The contribution of gut microbiota via the STING pathway to antitumor immune responses has also been observed in both hepatocellular carcinoma (HCC) patients and experimental HCC models (100).

Radiation therapeutic response is not solely influenced by the gut microbiome. A recent investigation using CRC mouse models revealed that modifications in oral microbiota led to shifts in bacterial makeup within CRC tumors while leaving adjacent peritumor tissues unaffected. Notably, the migration of *Fusobacterium nucleatum* from the oral cavity to the CRC site was observed, hindering the effectiveness of radiotherapy and impacting prognosis. The administration of metronidazole successfully countered the detrimental effects of oral microbiome alterations on CRC radiotherapy outcomes. Furthermore, the oral microbiota was found to correlate with radiation-induced intestinal damage through its influence on intestinal microbial communities (101).

One drawback of radiotherapy is the emergence of undesired side effects affecting various organs in the body, such as toxicity to the digestive or nervous systems. Around 90% of cancer patients undergoing pelvic radiotherapy experience gastrointestinal (GI) toxicity, including symptoms like bloody diarrhea and gastritis, with many linked to gut dysbiosis. Hence, the gut microbiome, pivotal in regulating digestive function, significantly influences gastrointestinal ADRs to radiation. In a preclinical investigation, radiation-induced damage to intestinal villi height and mucosal thickness was observed, along with induced neural inflammation and cell death (102). Intriguingly, altering the gut microbiota effectively mitigated toxicity in both the gastrointestinal and neural systems, suggesting a key to the challenge of radiotherapy. A study conducted on gynecologic cancer patients yielded similar findings, demonstrating that modifying the vaginal microbiota resulted in changes to radiation-induced vaginal toxicities, including pain, dyspareunia, and sexual dysfunction (103).

## 4 Perspectives for microbiome-targeted solutions to improve cancer treatment outcomes

Understanding the significant influence of the various microbiota on the development, metastasis, and response to treatment of tumors not only allows the use of microbiome components as biomarkers for selecting appropriate treatment methods, achieving high efficacy with minimized toxicity but also opens perspectives for intervening to alter the microbiome to bring about favorable outcomes for cancer patients. The advantages of microbiome-targeted interventions lie in their high feasibility, as they do not require overly advanced, costly methods, are minimally invasive, have fewer long-term systemic side effects, and are non-irreversible for patients. Another particularly notable aspect is that interventions targeting the microbiome can be personalized according to the unique microbiome characteristics of each patient. Additionally, microbiome-targeted interventions can be combined with various therapeutic modalities in a personalized manner to maximize benefits for patients. The followings are primary strategies for microbiome interventions.

### 4.1 Diet and supplement-based interventions

Substantial evidence highlights significant differences in microbiota composition, beneficial/pathogenic microbe abundance, and metabolite profiles between healthy individuals and cancer patients, as well as among cancer patients with varied treatment responses (63). Consequently, modifying dietary habits to promote beneficial microbe growth and diminish harmful ones can positively influence treatment outcomes in cancer patients. Recognized as pivotal components of cancer precision medicine, diet, and supplement-based interventions target specific dietary factors and nutritional supplements to optimize treatment efficacy. For example, embracing a diet abundant in fiber, fruits, vegetables, and fermented foods fosters a diverse and healthy microbiota. Additionally, a proactive approach to microbiome influence involves supplementing beneficial microbes and their substrates through microbiome modulators such as probiotics, prebiotics, and postbiotics to elicit desired effects. This notion finds support in numerous clinical studies across diverse cancer types (**Table 2**).

**Table 2:** Clinical evidence supporting diet and supplement-based interventions for better cancer treatment outcomes.

Cancer type	Sample size	Treatment method	Microbiome Intervention	Main findings	Ref
Multiple types	20	CT	IF	Reduce DNA damage in leukocytes. Decrease IGF-1 levels	(104)
Multiple types	6	CRT	KD	Tumor regression occurred in 5 of 6 patients. Once KD ended, their disease progressed rapidly	(105)
GC	120	CT	NI	NI was associated with significantly better treatment prognoses	(106)
PaC	19	ST	KD	KD is a safe adjuvant nutritional intervention in PaC treatment	(107)
OC, UC	45	CT	KD	Decrease cancer related growth factors	(108)
BC	60	CT	KD	Improve overall survival with no substantial side effects	(109)
PaC	30	ST	KD	Revert some cancer metabolite biomarkers	(110)
PC	42	ADT	KD	No change in prostate specific antigen and high-sensitivity C-reactive protein	(111)
Melanoma	438	IMT	HF and probiotics	Improve progression-free survival	(88)
LC	39	CT and IMT	HF	Better clinical outcomes. Enrichment of beneficial gut bacteria. Increase propionate level, which correlate with longer overall survival	(112)
RC	30	IMT	Probiotics: Bifidogenic live bacterial product (CBM588)	Increase progression-free survival and response rate	(113)
BC	159	CT	Probiotics	Prevent CT-related cognitive impairment	(114)
CRC	46	CT	Probiotics	Reduce the incidence and severity of gastrointestinal toxicity	(115)
Pelvic cancer	229	RT	Probiotics: Bifilact(®)	Reduce RID	(116)
Multiple types	206	RT	Probiotics: *Lactobacillus rhamnosus* (Antibiophilus)	Higher benefit/risk ratio Antibiophilus group	(117)
CC	54	RT	Probiotics: *Lactobacillus acidophilus* LA-5 and *Bifidobacterium animalis* subsp. lactis BB-12	Reduce RID	(118)
CRC	140	CT	Probiotics: *L. acidophilus* BMC12130, *L. casei* BCMC12313, *L. lactis* BCMC12451, *B. bifidum* BCMC02290, *B. longum* BCMC02120 and *B. infantis* BCMC02129	Improve quality of life Reduce certain inflammatory biomarkers and side effects	(119)
Multiple types	100	CT	Probiotics: *B. infantis*, *L. acidophilus*, *E. faecalis* and *Bacillus cereus*	Effectively and safely treat functional constipation during CT	(120)
CRC	143	CT	Probiotics*: L. rhamnosus* GG ATCC 53103	Reduce diarrhea side effects	(121)
CRC	150	CT	Probiotics*: L. rhamnosus* GG ATCC 53103	Reduce 5-FU-based CT-related diarrhea	(122)
HNC	75	RT	Probiotics: *L. brevis* CD2	No efficacy in preventing radiation-induced mucositis	(123)
NC	99	CRT	Probiotics: *B. longum*, *L. lactis* and *E. faecium*	Enhance immune response. Reduce severity of oral mucositis	(124)
HNC	200	CRT	Probiotics: *L. brevis* CD2	Reduce the incidence of severe oral mucositis	(125)
HNC	86	RT	Probiotics: *L. acidophilus*, *L. rhamnosus*, *B.* *longum* and *Saccharomyces boulardii*	Reduce oral *Candida* spp.	(126)
LC	95	CT	Probiotics: *L. casei* LC9018	Useful agent for the treatment of cancer and prevent pleural effusions	(127)
PaC	101	ST	Synbiotics: *Lactobacillus casei* strain Shirota, *Bifidobacterium breve* strain Yakult and GOS	Reduce postoperative infectious complications	(128)
CRC	100	ST	Probiotics: *Lactobacillus plantarum* (CGMCC No. 1258), *Lactobacillus acidophilus* (LA-11) and *Bifido-bacterium longum* (BL88)	Improve gut mucosal barrier integrity Reduce infectious complications	(129)
CRC	60	ST	Probiotics: *Bifidobacterium longum*, *Lactobacillus acidophilus*, and *Enterococcus faecalis*	Reduce the short-term infectious complications	(130)
CRC	150	ST	Probiotics: *Lactobacillus plantarum* (CGMCC no.1258), *Lactobacillus acidophilus*-11 and *Bifidobacterium longum*-88	Reduce the rate of postoperative septicemia	(131)
CRC	75	ST	Synbiotics: *Pediococcus pentosaceus* 5-33:3, *Leuconostoc mesenteroides* 32-77:1, *Lactobacillus paracasei* ssp. paracasei 19, *Lactobacillus plantarum* 2362 and beta-glucan, inulin, pectin and resistant starch	Postcolectomy gastrointestinal function may benefit	(132)
CRC	91	ST	Synbiotics: *Lactobacillus acidophilus*, *Lactobacillus rhamnosus*, *Lactobacillus casei*, *Bifi dobacterium* and FOS	Reduce postoperative infection rates	(133)
Periampullary cancer	54	ST	Synbiotics: *Lactobacillus acidophilus* 10, *Lactobacillus rhamnosus* HS 111, *Lactobacillus casei* 10, *Bifidobacterium bifidum* and FOS	Reduce postoperative mortality and complication rates	(134)
NC	77	CRT	Probiotics: *L. plantarum* MH-301, *B. animalis* subsp. Lactis LPL-RH, *L. rhamnosus* LGG-18 and *L. acidophilus*	Reduces the severity of oral mucositis by enhancing the immune response and modifying the structure of gut microbiota	(135)
Acute leukemia	60	CT	Prbiotics: *Lactobacillus rhamnosus* GG	Reduce CT-induced gastrointestinal side effects	(136)
LC	41	CT	Probiotics: *Clostridium butyricum*	Reduce CT-induced diarrhea Reduce systemic inflammatory response	(137)
CRC	70	ST	Probiotics: Two combined live bacteria	Reduce the incidence of diarrhea and abdominal distension Promote the recovery of intestinal function	(138)
CRC	15	ST	Probiotics: *Bifidobacterium* *lactis* Bl-04 (ATCC SD5219), *Lactobacillus acidophilus*	Probiotics have potential therapeutic benefits in CRC	(139)
CC, CRC	482	RT	Probiotics: Four strains of *Lactobacilli*, three strains of *Bifidobacteria* and one strain of *Streptococcus*	Prevent risk of RID	(140)
Advanced solid tumors	40	IMT	Probiotics: 30-species microbial consortium (Microbial Ecosystem Therapeutic 4, MET4)	Probiotics is potential to use as a therapeutic co-intervention with IMT	(141)
CRC	52	ST	Probiotics: *Lactobacillus acidophilus*, *Lactobacillus lactis*, *Lactobacillus casei* subsp, *Bifidobacterium longum*, *Bifidobacterium bifidum* and *Bifidobacterium infantis*	Reduce pro-inflammatory cytokines (except for IFN-gamma)	(142)
CC	70	CRT	Synbiotics: *L. acidophilus*, *B. lactis* and inulin	Reduce fecal calprotectin levels and the frequency/intensity of vomiting side effect	(143)
Multiple types	46	RT	Synbiotics: *S. thermophiles*, *Lactobacilli*, *Bifidobacter* and honey	Reduce the incidence of RID and the use of antidiarrheal medication	(144)
CC	20	CRT	Prebiotics: hydrolysed rice bran	Relieve diarrhea side effect	(145)
CC	100	RT	Prebiotics: resistant starch	No significant benefit	(146)
PC, GC	60	RT	Prebiotics: psyllium	Psyllium was an effective method to control RID	(147)
GC	38	RT	Prebiotics: Inulin and FOS	Improve quality of life	(148)

BC, Breast cancer; LC, Lung cancer; CC, Cervical cancer; CRC, Colorectal canscer; HNC, Head and neck cancer; PC, Prostate cancer; GC, Gynaecological cancer; PaC, Pancreatobiliary cancer; UC, Uterus Cancer; NC, Nasopharyngeal cancer; GC, Gastrointestinal cancer; OC, Ovarian cancer; RC, Renal cancer; CRT, Chemoradiotherapy; CT, Chemotherapy; RT, Radiotherapy; IMT, Immunotherapy; ST, Surgical therapy; ADT, Androgen deprivation therapy; KD, Ketogenic diet; IF, Intermittent Fasting; NI, Nutritional intervention; HF, High fiber diet; 5-FU, 5-fluorouracil; RID, Radiation-induced diarrhea; GOS, Galacto-oligosaccharides; FOS, Fructo-oligosaccharide.

Despite increasing interest in these interventions, challenges persist regarding standardization, efficacy, and safety (149). Additionally, some contrary findings have been reported, in which, probiotics use compromised the efficacy of ICIs in cancer patients (150). Therefore, rigorous clinical trials are indispensable to assess the efficacy and safety of diet approaches, establish optimal dosages and formulations, and ascertain their compatibility with conventional cancer therapies. Furthermore, personalized strategies are imperative to tailor diet and supplement interventions to individual patient characteristics, including cancer type, stage, genetic profile, and lifestyle factors.

### 4.2 Fecal microbiota transplantation

Bacteriotherapy, which involves utilizing microbes or their byproducts to treat illnesses, encompasses various approaches. Alongside supplementing specific microbes through probiotics, Fecal Microbiota Transplantation (FMT) is emerging as a promising method to utilize the gut microbiome’s potential to modulate therapeutic responses and enhance patient outcomes. FMT entails transferring fecal material from a healthy or therapeutically responsive donor into the gastrointestinal tract of a patient to restore or manipulate the microbiota composition (151). According to a Europe-wide survey conducted in 2019, 31 FMT centers across 17 countries performed a total of 1,874 procedures. However, the sole officially approved indication for FMT remains *Clostridioides difficile* infection (152).

Despite accumulating evidence demonstrating the potential benefits of FMT in enhancing outcomes of cancer treatment in experimental models, its application in cancer patients is currently limited to research and clinical trials (**Table 3**). Often integrated with other therapeutic approaches, some of these trials have shown promising results, suggesting a potential avenue for effective cancer treatment. FMT has been demonstrated to enhance the efficacy of immunotherapy by bolstering anti-tumor immune responses in CRC and melanoma (156, 195). Additionally, FMT has proven effective in mitigating treatment-related toxicities, such as chemotherapy-induced gastrointestinal symptoms (194). In particular, several clinical studies have demonstrated the efficacy of FMT in treating Gastrointestinal Acute Graft-versus-Host Disease (GI-aGvHD), a severe and potentially life-threatening complication arising from Allogeneic Stem Cell Transplantation (allo-SCT), an advanced therapeutic approach utilized in the management of hematologic malignancies.

**Table 3:** Application of fecal microbiota transplantation in cancer clinical settings.

Cancer Type	Recruiting Patients	Intervention/Treatment	Status/Findings	Ref
CRC	MSS-mCRC patients	FMT from anti-PD1 responders via stool capsules plus Tislelizumab and Fruquintinib	FMT plus Tislelizumab and Fruquintinib as third-line or beyond treatment demonstrated enhanced survival rates and manageable safety in refractory MSS-mCRC, suggesting a promising treatment option for this patient population.	(153)
CRC	Anti-PD-1 Non-responders Metastatic Colorectal Cancer	FMT from PD-1 responding mismatch-repair deficiency (dMMR) CRC patients via colonoscopy followed by stool capsules	Active, not recruiting. To assess the effectiveness of combining pembrolizumab or nivolumab with FMT obtained from PD-1 responding dMMR-CRC patients for treating PD-1 non-responding dMMR CRC patients.	(154)
CRC	CRC patients with advanced stages	FMT from responder donors plus Sintilimab and Fruquintinib	Recruiting. To evaluate the effectiveness and safety of combining FMT plus Sintilimab and Fruquintinib as the later line treatment option for advanced-stage CRC.	(155)
Melanoma	A anti-PD-L1-refractory metastatic melanoma patient (a case report)	FMT from anti-PD-L1 responders via colonoscopy + Pembrolizumab	After FMT, the patient displayed a reduced presence of subcutaneous disease. Although there was a recurrence in the small bowel that required resection, the patient continued Pembrolizumab treatment, and as of the current writing, there is no sign of melanoma recurrence.	(156)
Melanoma	Untreated patients with advanced melanoma	FMT from healthy donors plus Pembrolizumab or Nivolumab	Active, not recruiting. No grade 3 adverse events were documented from FMT alone. The ORR was 65% (13/20), with 20% (4) achieving CR. Responders witnessed an increase in immunogenic and a decrease in harmful bacteria after FMT. FMT from healthy donors appears to be safe in the first-line treatment context.	(157)
Melanoma	αPD1-refractory patients with advanced stage cutaneous melanoma	FMT from ICI-R or ICI-NR metastatic melanoma donors	Recruiting. To explore whether transferring the microbiota of either ICI-R or ICI-NR patients through FMT can alter the immunotherapy response in patients with refractory metastatic melanoma.	(158)
Melanoma	Anti-PD-1-refractory metastatic melanoma	FMT from anti-PD1 responders via colonoscopy plus Pembrolizumab	Active, not recruiting. FMT combined with anti-PD-1 treatment induced changes in the gut microbiome composition and transformed the tumor microenvironment, effectively overcoming resistance to anti-PD-1 in a specific group of advanced melanoma patients.	(159)
Melanoma	Anti-PD-1-refractory metastatic melanoma	FMT from ICIs responders via colonoscopy followed by stool capsules	Unknown status. Clinical responses were observed in three patients, with two PR and one CR. Importantly, FMT treatment correlated with beneficial alterations in immune cell infiltration and gene expression profiles in both the gut lamina propria and the tumor microenvironment.	(160)
Melanoma	Patients with unresectable or metastatic melanoma naïve for both anti-CTLA-4 and anti-PD1/PDL-1 inhibitors	FMT from healthy donors via stool capsules (MaaT013) plus anti-ipilimumab and nivolumab	Recruiting. To assess the potential to improve the response to a combination of anti-CTLA-4 and anti-PD-1 while ensuring the safety profile of these medications.	(161)
Melanoma, NSCLC	Melanoma and NSCLC metastatic	FMT from healthy donors via stool capsules	Active, not recruiting. To evaluate the effectiveness of FMT in anti-tumor activity of FMT when administered in combination with ICIs therapy.	(162)
Melanoma, NSCLC	Refractory or inoperable melanoma, MSH-H, dMMr or NSCLC	FMT from ICIs durable CR donors via stool capsules plus Nivolumab	Unknown status. To assess the safety and effectiveness of combining FMT with Nivolumab.	(163)
NSCLC	Stage III/V NSCLC naïve for PD/PDL1 inhibitors	FMT from greater response to immunotherapy or not, in combination with the PDL/PDL1 agent	Recruiting. To assess the safety of FMT and the treatment response.	(164)
NSCLC	Advanced or metastatic NSCLC	FMT	Unknown status. To assess the safety of combining FMT with PD-1/PD-L1 Monoclonal Antibodies in the treatment of advanced NSCLC, and analyze the impact of FMT on intestinal flora and immunophenotype of patients.	(165)
RC	Advanced Renal Cell carcinoma	FMT from ICIs responders via colonoscopy followed by stool capsules plus Pembrolizumab and Axitinib	Active, not recruiting. To evaluate the improving response rates to ICIs.	(166)
RC	Metastatic renal cell carcinoma	FMT from healthy donors	Active, not recruiting. Incorporating FMT into ICI therapy showed a safety profile in unselected 1L mRCC patients and yielded promising clinical efficacy results.	(167)
SC	mGC, ESCC, HCC refractory to anti-PD-(L)1 inhibitors	FMT from CR or PR donors treated with nivolumab or pembrolizumab monotherapy	Unknown status. FMT with potent microbiota has the potential to overcome resistance to anti-PD-1 inhibitors by altering the tumor microenvironment in advanced SC.	(168)
SC	Advanced, unresectable, or metastatic SC patients during anti-PD-(L)1 therapy.	FMT with Nivolumab	Not yet recruiting. To assess both the efficacy and safety of combining FMT with nivolumab in patients diagnosed with advanced, unresectable, or metastatic SC who have experienced disease progression during anti-PD-(L)1 therapy.	(169)
GC	Anti-PD-1 refractory GI cancers	FMT from healthy donors via capsule + Nivolumab	Active, not recruiting. FMT plus anti-PD-1 may overcome the resistance to anti-PD-1 against GI cancer via changing gut microbiota structure	(170)
GC	A gastric adenocarcinoma metastatic patient treated by Pembrolizumab had ICI-associated colitis (A case report)	FMT from healthy donors via colonoscopy	After FMT, the symptoms associated with colitis decreased and he was discharged with a steroid taper. He died 1 month after FMT due to cancer but without recurrent colitis.	(171)
Genitourinary cancer	Genitourinary Cancer treated by ICIs with severe IMC	FMT from healthy donors	Using FMT as a first-line treatment option may represent a safe and effective steroid sparing alternative to the current standard treatment for IMC.	(172)
PC	Metastatic castration-resistant prostate cancer	FMT from pembrolizumab responder donors	Recruiting. To investigate the anticancer potential of FMT from patients who respond to pembrolizumab into those who have not responded in metastatic castration-resistant prostate cancer patients.	(173)
Mesothelioma	Metastatic Mesothelioma	FMT from a healthy family donor via colonoscopy plus Pembrolizumab	Completed. To optimize the gut microbiome through FMT to augment the effectiveness of Pembrolizumab.	(174)
HC, SC	Hematological malignancies and solid tumors	FMT from healthy donors via colonoscopy with universal stool plus Bezlotoxumab (4/19 patients)	FMT is a safe and effective treatment for recurrent CDI in cancer patients and provides rapid resolution of symptoms.	(175)
SCLC, mRC	A small lung cancer and a metastatic renal cell carcinoma patients with refractory ICI-associated colitis (Case reports)	FMT from healthy donors via colonoscopy	Offered more compelling proof that FMT helped a lasting reduction in steroid dependency for IMC, therefore the patient can reclaim the advantages of resuming ICI therapy, leading to enhanced cancer prognosis.	(176)
PC, Genitourinary cancer	A metastatic urothelial carcinoma and a prostate cancer with ICI-associated colitis (Case reports)	FMT from healthy donors	After FMT, there was a restoration of the gut microbiome and a relative increase in the proportion of regulatory T-cells within the colonic mucosa.	(177)
Malignancy	Any malignancy treated with cancer immunotherapy	FMT from ICIs responders via colonoscopy	Recruiting. To demonstrate the feasibility of this FMT approach as a novel option in any malignancy patients undergoing immunotherapy.	(178)
HC	AML, Lymphoma, MDS, MM, MPN patients had steroid-resistant or steroid-dependent lower GI-aGvHD	FMT from healthy donors via capsules	FMT was generally well-tolerated. Following FMT, there was augmentation of beneficial Clostridiales and a reduction in pathogenic Enterobacteriales.	(179)
HC	Patients had steroid-resistant or steroid-dependent GI-aGvHD grade III-IV after allo-SCT	FMT from healthy donors via stool capsules (MaaT013)	The delivery of FMT was deemed safe in severely immunocompromised patients, with observed positive responses in certain individuals suffering from GI-aGvHD.	(180)
HC	AML patients undergoing intensive chemotherapy or allo-SCT	FMT via standardized oral capsules	Third-partyFMT was found to be safe and improved intestinal dysbiosis for allo-SCT and AML recipients. However, it did not lead to a reduction in infections.	(181)
HC	Patients undergoing a myeloablative allo-HSCT	FMT from healthy donor	Currently undergoing the analysis phase, assessing outcomes one year post-FMT. To evaluate the effectiveness of FMT in preventing complications associated with allo-SCT, focusing specifically on GvHD.	(182)
HC	AML, SAA, MDS, HAL patients had intestinal steroid-refractory aGVHD after SCT	FMT from healthy donors plus Ruxolitinib	The ORR, DOR, OS, EFS were positive. GVHD relapse rate was 33.3% in responders. The diversity of the intestinal microbiota increase in responders. FMT with Ruxolitinib could be an effective treatment for these specific patients.	(183)
HC	AML, AA, MDS, CML and other hematologic disease patients had steroid-refractory GI-GvHD after SCT	FMT from healthy donor via NJ or gastric tube	Within the follow-up period, the FMT group showed a better OS, and higher EFS time compare to control group. The mortality rate was lower in the FMT group. FMT may serve as a therapeutic option for grade IV steroid-refractory GI-GvHD.	(184)
HC	AML, MDS, T-PLL and Thalassemia patients had steroid refractory GvHD after allo-SCT	FMT from healthy donor via NJ tube or cryoconserved capsules	Positive effects on steroid-refractory were noted after FMT without the occurrence of major adverse events. Stool frequencies and volumes reduced after FMT, alongside noticeable attenuation of both grading and staging of steroid-refractory GvHD.	(185)
HC	AML, MDS, MPD, Hodgkin’s lymphoma, or non-Hodgkin’s lymphoma had steroid-refractory or steroid-dependent, acute or late-onset aGvHD after allo-SCT	FMT from healthy donors via NJ	Durable remission of steroid-refractory or steroid-dependent GvHD after FMT correlated with enhanced survival rates after FMT. FMT is a promising potential as a therapy for steroid-refractory or steroid-dependent GvHD.	(186)
HC	AML, ALL, MDS, CML, HAL had steroid refractory GI-aGvHD after allo-SCT	FMT from healthy donors via nasoduodenal	Following FMT, all patients experienced relief from clinical symptoms, an enrichment of beneficial bacteria and reconstruction of microbiota composition. In comparison to the non-FMT group, FMT patients exhibited a higher PFS. Thus, FMT emerges as a therapeutic option for GI-aGVHD	(187)
HC	Two AML and one MDS patients had severe refractory GI-aGvHD after allo-SCT	FMT from healthy donors	All three patients demonstrated clinical improvement after FMT with reduced stool volumes that normalized with repeated interventions. Altering the intestinal microbiota by FMT is an appealing and innovative treatment strategy for patients with refractory GI-aGvHD.	(188)
HC	AML patients had steroid-resistant or steroid-dependent gut aGvHD	FMT from healthy donor via infusion of a fecal suspension	FMT was safely administered to patients with AML undergoing SCT and could potentially provide a new therapeutic avenue for aGVHD.	(189)
HC	Patients had steroid-resistant or steroid-dependent GI-aGvHD after allo-SCT	FMT from healthy donors	Unknown status. To assess the safety and feasibility of using frozen capsules containing fecal microbiota from healthy donors as a treatment for steroid-resistant or steroid-dependent GI-aGvHD.	(190)
HC	Patients had steroid-resistant or steroid-dependent GI-aGvHD grade III-IV after allo-SCT	FMT from healthy donors via NJ tube	Unknown status. To assess the safety and effectiveness of FMT as a treatment for GI-aGvHD. FMT shows promise as a potentially beneficial intervention in this challenging clinical scenario.	(191)
HC	Patients designated to allo-SCT	FMT from healthy donor via capsules	Terminated. To estimate the safety and efficacy of FMT administered through oral capsules compared to placebo capsules.	(192)
HC	AML patients treated with intensive chemotherapy and antibiotics	Autologous FMT	The use of autologous FMT seems to be safe and shows potential effectiveness in restoring gut microbiota, achieving excellent reconstruction based on richness and diversity indices at the species level.	(193)
HC, SC	Underlying hematologic or solid malignancies patients undergoing with cytotoxic chemotherapy that recur CDI	FMT from healthy donor via colonoscopy with frozen stool	FMT represents a highly effective and safe treatment choice for cancer patients undergoing cytotoxic chemotherapy experiencing multiple recurrences of CDI.	(194)

1L mRCC, First-line metastatic renal cell carcinoma; aCRC, Advanced Colorectal Cancer; ALL, Acute lymphoblastic leukemia; Allo-HSCT, Allogeneic haematopoietic stem-cell transplantation; AMT, Acute myeloid leukaemia; Anti-PD-1, Anti-programmed cell death protein 1; Anti-PD-L1, Anti-programmed death-ligand 1; aRCC, Advanced Renal cell carcinoma; BM, Bowel movements; CDI, Clostridium difficile infection; CML, Chronic myeloid leukemia; CR, Complete responses; CR; PR, Complete response; partial response; CRC, Colorectal Cancer; CTLA-4, Cytotoxic T-Lymphocyte-Associated protein 4; dMMR, Mismatch-repair deficiency; DOR, Durable overall response; EFS, Event-free survival; ESCC, Esophageal squamous cell carcinoma; FMT, Fecal microbiota transplantation; GC, Gastrointestinal cancer; GI, Gastrointestinal; GI-aGvHD, Gastrointestinal acute graft-versus-host disease; HAL, Hybrid acute leukemia; HCC, Hepatocellular carcinoma; HC, Hematologic cancer; ICI, Immuno checkpoint inhibitor; ICI-NR, ICI-non-responding; ICI-R, ICI-responding; IMC, Immune-mediated colitis; irAE, Immune-related adverse events; MaaT013, Pooled allogeneic faecal microbiota; MDS, Myelodysplastic syndrome; mGC, Metastatic gastric cancer; MM, Multiple myeloma; MPD, Myeloproliferative disorder; MPN, Myeloproliferative neoplasms; MSH-H, Microsatellite instability-high; MSS-mCRC, Microsatellite stable-Metastatic colorectal cancer; NJ, Nasojejunal; NSCLC, Non-Small Cell Lung Cancer; ORR, Objective response rate; OS, Overall survival; PFS, Progression-free survival; RC, Renal cancer; SAA, Severe aplastic anemia; SCLC, Small cell lung cancer; SCT, Stem cell transplant; Solid cancer, SC.

However, challenges and considerations accompany the implementation of FMT in cancer treatment. These include standardizing donor selection and screening procedures, optimizing FMT protocols, determining optimal timing and dosing, and managing potential risks such as infection transmission and immune-related adverse events. That is the reason why FMT has not been approved for use in clinical setting, except for *Clostridioides difficile* infection, in European countries. Despite these challenges, FMT represents a promising avenue for precision oncology, offering a personalized and microbiome-based approach to cancer therapy. Further research is necessary to elucidate the mechanisms of action, optimize treatment protocols, and identify patient subgroups most likely to benefit from FMT. Overall, FMT holds potential as an innovative strategy to complement existing cancer treatment modalities and improve outcomes for cancer patients.

### 4.3 Antibiotic-based interventions for microbiome

Antibiotics, traditionally used to combat bacterial infections, have garnered attention for their potential to influence cancer treatment responses through modulation of the microbiome through both preclinical and clinical research (196). Despite promising findings in preclinical research indicating potential benefits of antibiotics in enhancing treatment efficacy and reducing adverse reactions in cancer therapy, clinical studies across diverse cancer types consistently demonstrate that antibiotic usage before or during treatment is associated with worsened outcomes, notably in immunotherapy (**Table 4**). These observations suggest an additional strategy for regulating microbiota in cancer precision medicine through the selective use of antibiotics given the requirement for thorough research for the appropriate antibiotic. Targeting harmful microbes with antibiotics to manipulate microbial communities can optimize treatment responses and reduce adverse effects. However, careful consideration, especially in antibiotic dosage is necessary to avoid disturbing the beneficial microbiota, leading to clinical complications (209) and the danger of antibiotic resistance (210).

**Table 4:** Microbiota-mediated impacts of antibiotics on anti-cancer treatment.

Cancer type	Treatment method	Outcome affected	Ref
NA	RT	Vancomycin enhanced the antitumor immune response triggered by RT and inhibited tumor growth by modulating butyrate-producing bacteria.	(70)
NA	CT	Treatment with antibiotics hampers the adverse drug reactions induced by paclitaxel chemotherapy.	(197)
NA	RT	Vancomycin reduced the presence of gut bacteria responsible for producing butyrate and amplified the body's immune response against tumors when combined with ionizing radiation (IR).	(99)
LiC	ICI	Antibiotics was linked to poorer outcomes	(198)
GC	ICI	Previous administration of antibiotics consistently correlated with reduced survival following ICI treatment, whereas it did not impact outcomes in patients treated with irinotecan.	(199)
RC, NSCLC	ICI	Antibiotics was link with poor clinical benefits of ICI	(200)
NSCLC	ICI	Antibiotics was associated with inferior PFS and OS	(201)
Multiple types	ICI	Exposure to any antibiotic, particularly fluoroquinolones, within one year prior to ICI, was linked to poorer OS	(202)
Melanoma, NSCLC	ICI	Early administration of antibiotics served as an independent poor prognostic factor in NSCLC patients treated with anti-PD-1/L1, but not in melanoma patients.	(203)
Malignancy	ICI	The utilization of antibiotics during ICI markedly diminished the effectiveness of treatment	(204)
NA	CT	Disruption of the microbiome caused by antibiotics exacerbated chemotherapy-induced diarrhea.	(205)
PaC	CT	Incorporating antibiotics into first-line gemcitabine chemotherapy regimens could potentially enhance outcomes.	(206)
OC	CT	Treatment with antibiotics was linked to reduced PFS and OS	(207)
NSCLC	ICI	The utilization of antibiotics within 21 days before and after the initiation of anti-PD-1 treatment significantly decreased OS and PFS	(208)

NA, Not avaiable; GC, Gastrointestinal cancer; LiC, Liver cancer; OC, Ovarian cancer; PaC, Pancreatobiliary cancer; NSCLC, Non-Small Cell Lung Cancer; RC, Renal cancer; RT, Radiotherapy; CT, Chemotherapy; Immuno-checkpoint inhibitor, IC.

### 4.4 Modulation of other local microbiotas beyond the gut

Local interventions targeting microbiotas beyond the gut microbiota are emerging as promising strategies in cancer precision therapy, aiming to harness the influence of various microbial communities on tumor biology and treatment responses. While much attention has been focused on the gut microbiota, other microbiotas throughout the body, including those in the oral cavity, skin, respiratory tract, urogenital tract, and tumor microenvironment, also play significant roles in cancer development and treatment.

Research has revealed the intricate interactions between these microbiotas and cancer, highlighting their potential as therapeutic targets for precision therapy (15, 16). Local interventions seek to modulate the composition and function of these microbiotas to enhance treatment efficacy, reduce treatment-related toxicities, and improve patient outcomes. These interventions encompass a range of approaches, including probiotics, prebiotics, antibiotics, microbial metabolites, targeted therapies, and physical interventions.

In the oral cavity, interventions targeting the oral microbiota, such as mouthwash formulations containing probiotics or antimicrobial agents, hold promise for preventing oral mucositis and reducing the risk of secondary infections in patients undergoing radiation therapy or chemotherapy for head and neck cancers (101). Similarly, interventions targeting the skin microbiota may involve topical applications of probiotic formulations or antimicrobial agents to alleviate radiation-induced dermatitis and enhance wound healing in patients with skin cancers (52, 87).

In the respiratory tract, interventions may include inhalation therapies with probiotics or antimicrobial agents to improve treatment responses and reduce the risk of respiratory infections in patients with lung cancers (211). Likewise, interventions targeting the urogenital microbiota may involve the use of vaginal probiotics or antimicrobial agents to prevent urinary tract infections and enhance treatment tolerability in patients with genitourinary cancers (76, 212).

Moreover, interventions targeting the tumor microenvironment may encompass immunomodulatory therapies, such as ICI or adoptive cell therapies, aimed at modulating the local immune response and tumor growth in various cancer types. Additionally, microbial-based therapies, such as oncolytic viruses or bacteria engineered to target tumors, offer novel strategies for directly targeting tumor cells and modulating the tumor microenvironment (213).

Despite the potential benefits of local interventions on non-gut microbiotas for cancer precision therapy, challenges exist in their implementation and optimization. These include the need for further research to elucidate the complex interactions between microbiotas and cancer, as well as the development of targeted and personalized interventions tailored to individual patient characteristics and tumor biology.

## 5 Conclusion

The field of pharmacomicrobiomics holds immense promise for revolutionizing precision cancer therapy by leveraging the intricate interplay between the microbiome and drug response. From preclinical investigations elucidating molecular mechanisms to clinical trials evaluating patient outcomes, advancements in this field offer unprecedented opportunities to optimize treatment strategies tailored to individual patients. By harnessing the potential of pharmacomicrobiomics, we can enhance treatment efficacy, minimize adverse effects, and ultimately improve patient outcomes in the era of precision oncology. However, challenges such as standardization, validation, and clinical translation remain, underscoring the need for continued research and collaborative efforts across disciplines.
